# Pregnancy in 4 women with childhood-onset steroid-sensitive nephrotic syndrome

**DOI:** 10.1007/s13730-013-0087-9

**Published:** 2013-07-03

**Authors:** Osamu Motoyama, Kikuo Iitaka

**Affiliations:** 1 Department of Pediatrics, Toho University Medical Center, Sakura Hospital, 564-1 Shimoshizu, Sakura, Chiba 285-8741 Japan; 2 Naruse Renal Clinic, Tokyo, Japan

**Keywords:** Childhood-onset nephrotic syndrome, Steroid-sensitive nephrotic syndrome, Pregnancy, Frequent relapsing nephrotic syndrome

## Abstract

Four women with childhood-onset steroid-sensitive nephrotic syndrome (SSNS) had 5 pregnancies. Their age at onset of SSNS was between 4 and 10 years, and age at pregnancy was between 21 and 31 years. Three patients with frequent relapsing nephrotic syndrome (NS) continued to relapse after 20 years of age. Two of them had relapses during 6–32 gestational weeks of pregnancy and were treated with prednisolone (PSL) 10–45 mg/day. One patient delivered a normal baby on 2 pregnancies. Another developed superimposed preeclampsia and her infant showed asymmetrical type of intrauterine growth restriction (IUGR). There was no relapse during pregnancy in 2 patients, including 1 with frequent relapses, who had no relapse over 5 years preceding the pregnancy. In all four patients, normal renal function and complete remission were noted at the last follow-up. Their 5 infants were well at 1–7 years of age. Although hypertension, growth failure of the placenta, and IUGR of the baby may complicate the pregnancy, most pregnancies with SSNS seem to result in normal birth, even when relapses occur during pregnancy and are treated with PSL.

## Introduction

Few reports of pregnancy in women with childhood-onset steroid-sensitive nephrotic syndrome (SSNS) are available. According to earlier reports by Makker and Heymann [1], Murai et al. [2], and Kosino et al. [3], termination of pregnancy was recommended when nephrotic syndrome (NS) recurred in a pregnant mother, because of poor prognosis of the fetus. The clinical course of pregnancy in both childhood-onset SSNS and adulthood-onset SSNS seems to be similar [1, 3]. In this paper, 3 pregnancies which were complicated with relapse of SSNS resulted in live birth and favorable outcome in patients with SSNS. Progress of treatment for NS, obstetrical management, and perinatal care may change the management of pregnancy in patients with SSNS [4, 5].

## Case report

Four women with SSNS conceived 5 babies. Table 1 shows the profiles of the 4 mothers with SSNS and their newborn babies. NS was diagnosed in patients who had heavy proteinuria (more than 40 mg/m^2^ per hour) and hypoalbuminemia (serum albumin <2.5 g/dL). Patients who responded during 8 weeks of prednisolone (PSL) treatment were defined as SSNS. Relapse was defined as a reappearance of proteinuria (2+ or greater by dipstick for 3 consecutive days). Frequent relapses were defined as two or more relapses within the first 6 months after initial response or four or more relapses during any 12-month period [6]. Estimated glomerular filtration rate was calculated by method of Matsuo et al. [7]. Their age at onset of NS was between 4 and 10 years. Hypertension, hematuria, or renal failure was not noted in these 4 patients, and the diagnosis of minor glomerular abnormalities was confirmed by renal biopsy in 3 patients (cases 2–4). The number of relapses of NS was between 4 and 77, and frequent relapsing NS was noted in 3 patients (cases 1–3). They were treated with PSL, combined with cyclophosphamide in 2 patients (cases 2 and 3), cyclosporine (CyA) in 3 (cases 1–3), and mizoribine in 1 (case 2). Cyclophosphamide at a dose of 2 mg/kg for 8–12 weeks and mizoribine at a dose of 100 mg/m^2^ were used before 14 years of age. Two patients (cases 3 and 4), who had no relapse for more than 5 years before pregnancy, had no relapse during pregnancy and had normal term deliveries. However, case 4 developed a fourth relapse 1 year after the delivery. Relapse occurred within 2 years prior to pregnancy in cases 1 and 2. The 2 patients had relapse during pregnancy. Case 1 had a relapse during her first and second pregnancies and case 2 had three relapses. Five infants were born alive. Four infants were delivered at term and were appropriate birth weights for their gestational ages, and one showed an intrauterine growth restriction (IUGR). In all four mothers, normal renal function and complete remission of NS were noted at the last follow-up, when they were between 24 and 33 years of age. Their 5 infants were well at 1–7 years of age and congenital malformation was not noted among them.

**Table 1:** Profiles of 4 mothers with childhood-onset steroid-sensitive nephrotic syndrome (SSNS) and their newborn babies

	Case 1	Case 2	Case 3	Case 4
Nephrotic syndrome				
Age at onset	10 years	4 years	5 years	4 years
Renal biopsy finding	ND	MCNS	MCNS	MCNS
Number of relapses	44	77	26	4
Treatment	PSL, CyA	PSL, CY, MZ, CyA	PSL, CY, CyA	PSL
Interval between pregnancy and the last relapse	1st: 9 months 2nd: 1 years	8 months	6 years	5 years
Numbers of relapses during pregnancy	1st: 1 2nd: 1	3	–	–
At delivery				
Age	1st: 26 years 2nd: 31 years	27 years	31 years	21 years
Gestational age	1st: 40 weeks 2nd: 38 weeks	38 weeks	40 weeks	40 weeks
Birth weight of infants	1st: AGA 2nd: AGA	SGA	AGA	AGA
At last follow-up				
Age	33 years	28 years	33 years	24 years
Blood pressure (mmHg)	120/70	122/78	110/70	110/70
Serum creatinine (mg/dL)	0.5	0.6	0.6	0.5
eGFR (mL/min/1.73 m^2^)	113	105	90	137
Proteinuria	–	–	–	–

*ND* not done, *MCNS* minimal change nephrotic syndrome, *PSL* prednisolone, *CyA* cyclosporine, *CY* cyclophosphamide, *MZ* mizoribine, *AGA* appropriate-for-gestational-age infant, *SGA* small-for-gestational-age infant, *eGFR* estimated glomerular filtration rate

Clinical findings, gestational weeks, and treatment for relapse of NS during pregnancy are shown in Tables 2 and 3.

**Table 2:** Clinical findings and treatment at relapse of nephrotic syndrome (NS) during pregnancy in case 1

	First pregnancy			Second pregnancy		
	Relapse		Delivery	Relapse		Delivery
Gestational weeks	10	20	40	7	22	38
Blood pressure (mmHg)	156/90	126/80	118/80	136/70	110/60	NA
Edema	+	−	−	−	−	−
Total protein (g/dL)	5.0	5.9	ND	6.5	ND	ND
Albumin (g/dL)	2.4	3.2	ND	3.6	ND	ND
Creatinine (mg/dL)	0.5	0.4	ND	0.6	ND	ND
Proteinuria^a^	3+	–	–	3+	–	–
Treatment						
PSL (mg/day)	20	10	10	45	10	10
Time to induce remission	4 days			6 days		

*NA* not available, *ND* not done, *PSL* prednisolone

^a^Degree of proteinuria detected by dipstick was graded as: 3+, 300 mg/dL

**Table 3:** Clinical findings and treatment at relapse of nephrotic syndrome (NS) during pregnancy in case 2

	First relapse		Second relapse		Third relapse		Delivery
Gestational weeks	6	8	22	28	32	36	38
Blood pressure v(mmHg)	144/90	128/80	130/84	134/80	134/86	140/90	142/92
Edema	−	−	−	−	−	−	+
Total protein (g/dL)	ND	ND	6.5	6.4	6.1	6.2	5.8
Albumin (g/dL)	ND	ND	3.1	3.0	2.3	2.8	2.5
Creatinine (mg/dL)	ND	ND	0.4	0.3	0.4	0.4	0.3
Proteinuria^a^	2+	−	3+	−	3+	3+	3+
Treatment							
PSL (mg/day)	20	12.5	30	17.5	20	20	20
CyA (mg/day)	150	125	75	75	50	25	0
Time to induce remission	1 weeks		3 weeks		10 weeks		

*ND* not done, *PSL* prednisolone, *CyA* cyclosporine

^a^Degree of proteinuria detected by dipstick was graded as: 2+, 100 mg/dL; 3+, 300 mg/dL

## Case 1

Case 1 had been treated with PSL 30 mg/day at the last relapse before the pregnancy. She was treated with PSL on alternate days with 5 mg at the onset of relapse during her first pregnancy. At relapse, when she was at 10 weeks of gestation during her first pregnancy, methylprednisolone 500 mg was administered intravenously, followed by oral PSL 20 mg/day. PSL was gradually tapered to 10 mg/day at 20 weeks of gestation and was maintained during pregnancy. Relapse during her second pregnancy occurred 5 months after PSL was discontinued. At relapse, when she was at 7 weeks of gestation, PSL was started at a dose of 45 mg/day. PSL was gradually reduced to 10 mg/day at 22 weeks of gestation and was maintained during pregnancy. Proteinuria disappeared within 1 week after the start of PSL in these relapses. After remission of NS, her blood pressure remained normal. The birth weights of infants and placenta (650 and 670 g, respectively) were normal in both deliveries.

## Case 2

Case 2 had been treated with PSL 20 mg/day at the last relapse before the pregnancy. She was treated with PSL 7.5 mg/day and CyA 150 mg/day (trough level 69–94 ng/mL) at 6 weeks of gestation. At relapse, she was treated with PSL 20 mg/day and proteinuria disappeared a week later. After PSL was reduced to 12.5 mg/day at 8 weeks of gestation, the second relapse occurred at 22 weeks of gestation. She was treated with PSL 30 mg/day. Proteinuria disappeared 3 weeks after the second relapse. After PSL was reduced to 17.5 mg/day at 28 weeks of gestation, the third relapse occurred. She had no edema and was treated with PSL 20 mg/day until delivery. After the third relapse at 32 weeks of gestation, proteinuria continued until delivery and her serum albumin was 2.5 g/dL at delivery. Fetal ultrasonography showed impaired fetal growth from 30 weeks of gestation and oligohydramnios after 36 weeks, but did not reveal any fetal malformations. She developed hypertension (142/92 mmHg) at bed rest at 38 weeks of gestation. Labor was induced by oxytocin injection and she delivered a female infant with an Apgar score of 9 and 9 at 1 and 5 min, respectively. Her body weight was 2171 g (below the 10th percentile for the Japanese population), body length was 41.7 cm (below the 10th percentile), and head circumference was 32.5 cm (above the 10th percentile). Small placenta (377 g) with no signs of chorioamnionitis or infarct of the placenta was noted. She had asymmetrical type of IUGR due to placental insufficiency and superimposed preeclampsia. After birth, she developed no complications and was discharged at 12 days of age. CyA was discontinued 2 weeks before delivery and breast-feeding was started after delivery. One month later, hypertension was resumed. Two months later, breast-feeding resumed. After delivery, 3 relapses occurred and CyA was restarted.

## Discussion

The effects of pregnancy on preexisting chronic kidney disease depend on the type of renal disease, the level of renal impairment, and the presence of hypertension. The prognosis of pregnancy in women with chronic kidney disease is thought to be good when their serum creatinine and blood pressure are normal [4]. Few reports of pregnancy in women with childhood-onset SSNS are available. Usually, relapse of SSNS during pregnancy does not occur after long-term remission prior to pregnancy [1, 3, 8]. Pregnancy dose not seem to be a trigger causing relapse of NS and does not affect the natural history of minimal change NS [9]. Favorable prognosis of pregnancy within remission of SSNS has been reported. On the other hand, maternal severe proteinuria or hypoproteinemia may result in small-for-gestational-age infants [9]. Pregnant women with chronic kidney disease administered immunosuppressive drugs present the problems of an increased incidence of abortion, preterm delivery, IUGR, and low birth weight infants [10]. Some reports [1–3] have recommended termination of pregnancy when NS recurred in a pregnant mother, because of poor prognosis of the fetus.

Immunosuppressive drugs cross the placental barrier and enter the fetal circulation. PSL crosses the placenta and 66 % of PSL is metabolized into inactive prednisone by the placental 11-beta-hydroxysteroid dehydrogenase [10]. There was no sign of adrenal insufficiency in the fetus [2, 11]. PSL or CyA may inhibit placental growth and placental insufficiency seemed to increase IUGR. CyA also crosses the placenta. A suppression of T, B, and NK cells in infants exposed to maternal CyA may continue up to 1 year of age. CyA is excreted into breast milk and breast-feeding is not recommended while taking CyA because of its immunosuppressive effect in infants. CyA was discontinued 2 weeks before delivery in case 2. Cyclophosphamide and mizoribine are contraindicated during pregnancy because of fatal toxicity [10].

In our patients who had long-term remission of SSNS, pregnancy did not lead to relapse. The Japanese Society of Nephrology recommends pregnancy in women with NS after a remission period lasting longer than 6 months after the treatment was discontinued. Women of childbearing age who continue to relapse frequently during adulthood, as in cases 1 and 2 in this study, do not achieve remission without treatment. Among 110 children with SSNS who were treated at Toho University Medical Center, Omori Hospital (Tokyo, Japan) and Sakura Hospital between 1972 and 2005, 7 patients continued to relapse after 20 years of age and one was 34 years old when he had the last relapse [12]. Obstetrical management and perinatal care has progressed since the earlier reports published between 1972 and 1985 [1–3]. Neonatal mortality of low birth weight infants (<2500 g) in Japan continues to decrease from 8.8 % in 1980 and 4.8 % in 1990 to 2.4 % in 2000 [13]. Although hypertension, growth failure of the placenta due to PSL and CyA, and IUGR due to hypoproteinemia [9] may be complicated in some cases, most women with SSNS seem to have successful pregnancies, even when they had relapse during pregnancy and were treated with PSL [4, 11]. Chen et al. [11] also reported favorable outcome of pregnancy in 6 women presenting NS with normal renal function during pregnancy and who were treated with PSL. Their renal biopsy showed lupus nephritis in 3 patients, mesangial proliferative glomerulonephritis in 2, and endocapillary proliferative glomerulonephritis in 1. They were treated with intravenous methylprednisolone 1 g for 3 consecutive days, followed by oral PSL 0.5 mg/kg/day for 8 weeks or oral PSL 1 mg/kg/day for 8 weeks after 20 weeks of pregnancy. Five term infants were appropriate weights for gestational ages and an infant who weighed 1900 g was small for gestational age. The clinical course of these 6 mothers after delivery was good.

Case 1 presented with edema at relapse during her first pregnancy. A high dose of methylprednisolone was used to achieve remission and to maintain fetal circulation, as early as possible. PSL 45 mg/day was started at relapse during her second pregnancy. At both relapses, she went into remission early and delivered a normal baby. Case 2 developed relapse without edema and was treated with PSL 20 mg/day. When superimposed preeclampsia occurred at the third trimester of pregnancy, close follow-up by obstetricians and induced labor at an appropriate period resulted in the successful delivery of a small-for-gestational-age infant. There is no protocol of treatment for relapse of SSNS during pregnancy. The dose of PSL should be determined individually to induce remission in each relapse during pregnancy according to the treatment of previous relapse.
